# Good 5-year outcomes after arthroscopic treatment for femoroacetabular impingement syndrome

**DOI:** 10.1007/s00167-019-05429-y

**Published:** 2019-04-10

**Authors:** Axel Öhlin, Mattias Ahldén, Ida Lindman, Páll Jónasson, Neel Desai, Adad Baranto, Olufemi R. Ayeni, Mikael Sansone

**Affiliations:** 1 Department of Orthopaedics, Institute of Clinical Sciences, Sahlgrenska Academy, University of Gothenburg, Gothenburg, Sweden; 2 Orkuhusid Orthopaedic Clinic, Reykjavik, Iceland; 3 Division of Orthopaedic Surgery, Department of Surgery, McMaster University, Hamilton, ON Canada; 4 Sahlgrenska Universitetssjukhuset Mölndal, Göteborgsvägen 31, 431 80 Mölndal, Sweden

**Keywords:** FAI syndrome, Hip arthroscopy, Outcome, Long term

## Abstract

**Purpose:**

The purpose of the present study was to evaluate the outcome of arthroscopic treatment for femoroacetabular impingement (FAI) syndrome 5 years post-surgery using patient-reported outcome scores (PROMs) validated for a young and active population with hip complaints.

**Methods:**

Patients were prospectively included in the study. A total of 184 patients [males = 110 (59.8%), females = 74 (40.2%)], with mean age 38.0, underwent arthroscopic treatment for FAI syndrome and were analysed. Preoperatively and at the 5-year follow-up, patients completed a set of self-administered web-based PROMs consisting of the International Hip Outcome Tool (iHOT-12), the Copenhagen Hip and Groin Outcome Score (HAGOS), the Hip Sports Activity Scale (HSAS), the EuroQoL-5 Dimension Questionnaire (EQ-5D), the EQ-Visual Analogue Scale (VAS) and the VAS for overall hip function and overall satisfaction. The Wilcoxon signed rank test was used to compare preoperative PROM values with those obtained at the 5-year follow-up.

**Results:**

A comparison of preoperative PROM scores and those obtained at the 5-year follow-up revealed statistically significant improvements for all outcome scores (*p* < 0.05), except for the HSAS score, which were unchanged; iHOT-12 (42.9 vs 67.2), HAGOS different subscales (50.2 vs 69.6, 55.7 vs 76.1, 59.2 vs 72.3, 41.1 vs 66.4, 30.8 vs 60.2, 31.6 vs 60.4), EQ-5D (0.570 vs 0.742), EQ-VAS (66.6 vs 74.4), HSAS (3.13 vs 3.17) and VAS for overall hip function (47.9 vs 69.2). At the 5-year follow-up, 154 patients reported that they were satisfied with surgery (84.6%). Survivorship at the 5-year follow-up was 86.4%.

**Conclusion:**

Arthroscopic treatment for FAI syndrome yields good patient-reported outcome at the 5-year follow-up.

**Level of evidence:**

II.

## Introduction

Femoroacetabular impingement (FAI) syndrome is a cause of hip pain and reduced range of motion (ROM) in young and active patients. FAI syndrome has furthermore been proposed as a cause of osteoarthrosis (OA) of the hip [1–4]. A prominent femoral head-neck junction (cam morphology) and/or a prominent acetabular rim (pincer morphology) causes mechanical conflict in the moving hip, which may also lead to damaged soft tissue in the joint [3].

Surgical treatment for FAI syndrome aims to reproduce normal hip anatomy, thereby reducing pain and improving hip function. Several recent studies have reported good results following arthroscopic treatment for FAI syndrome at short- to mid-term follow-ups [5–11]. The results of arthroscopic surgery have been reported to be similar to those of open surgery, regarding both clinical outcome measurements and the conversion rate to total hip arthroplasty (THA), although hip arthroscopy results in a significantly higher general health-related quality of life [12], with fewer complications [13].

As previously noted [14], there is a lack of studies evaluating the long-term outcome after arthroscopic treatment. As it is thought that cartilage overload can cause the degeneration of the articular surface, subsequently leading to OA, the long-term outcome is of particular interest in the treatment of FAI syndrome [15]. The amount of stress the articular surface is able to tolerate and the speed with which OA may develop are poorly understood.

The aim of the present study was to report outcomes at the 5-year follow-up after arthroscopic treatment for FAI syndrome, using patient-reported outcome measurements (PROM) developed for a young and active population. The primary hypothesis of the present study was that patients will experience a significant improvement at the 5-year follow-up.

## Materials and Methods

The study was conducted in agreement with the Helsinki Declaration. Informed consent was obtained from each patient in the study. Ethical approval for the study was granted by the Regional Ethical Review Board in Gothenburg at the Sahlgrenska Academy, Gothenburg University, Gothenburg, Sweden (registration number EPN 071-12).

Patients treated between 2011 and 2013 were included in this prospective cohort study. The follow-up took place 5 years postoperatively. The inclusion criterion was arthroscopic surgery for FAI syndrome. The diagnosis of FAI syndrome was based on patient history, physical examination and radiological findings consistent with FAI syndrome. The indication for surgery was an established diagnosis of FAI syndrome and failed non-surgical treatment. The contraindications for surgery included advanced OA (joint space < 2 mm) and severe dysplasia (lateral centre edge angle ≤ 20 degrees). The exclusion criterion was prior hip surgery. All the hip arthroscopies were performed at two centres by three orthopaedic surgeons.

There were 408 eligible patients, of which 47 patients were excluded due to prior hip surgery and 148 patients did not complete the 5-year follow-up. Twenty-nine patients (13.6%) converted to total hip arthroplasty during the follow-up period and were excluded. As a result, 184 patients were included for further analysis, 225 hips in total. The mean age was 38.0 (standard deviation (SD) ± 12.7) years and the youngest patient was 15 years of age. The demographic data are shown in Table 1.

**Table 1:** Patient demographics

Demographics	
Total number of patients	184
Total number of hips	225
Age—mean (SD)	38.0 (12.7)
Male/female (%)	110/74 (59.8/40.2)
Symptom duration—median (min–max)	24 (2–240) months
Operated side, right/left (%)	101/85 (54.3/45.7)

*SD* Standard deviation

Perioperative data were recorded at the time of surgery and included age, symptom duration, a description of cartilage status according to the classification by Konan et al. [16] and procedures performed. Preoperatively and at the 5-year follow-up, the patients completed a set of self-administered web-based PROMs consisting of the International Hip Outcome Tool (iHOT-12) [17], the Copenhagen Hip and Groin Outcome Score (HAGOS) [18], the Hip Sports Activity Scale (HSAS) [19], the EuroQoL-5 Dimension Questionnaire (EQ-5D) [20], the EQ-Visual Analogue Scale (VAS) [20] and the VAS for overall hip function. At the 5-year follow-up, the set of self-administered web-based PROMs also included a question asking whether or not the patients were satisfied with surgery. The EQ-5D was initially developed in Swedish. The iHOT-12, HAGOS and HSAS have previously been translated and culturally adapted to Swedish [21, 22]. Information regarding re-operations, including conversion to (THA), was recorded by the author from patient journals.

### Surgical technique

All the procedures were performed in an out-patient setting. The surgical technique has previously been described [23]. An antero-lateral portal and a mid-anterior portal were established with the patient in the supine position. Axial traction to the leg was applied to gain access to the central compartment of the hip joint for diagnostic overview, free-body removal and microfracture, as needed. Access to the peripheral compartment was achieved through a ligament-sparing interportal capsulotomy parallel to the fibres of the ileo-femoral ligament, with a minimal transverse cut to minimise the risk of iatrogenic instability [24, 25]. The capsulotomy is longitudinal and is, therefore, not closed. Acetabular over-coverage (pincer morphology) was resected using an “over-the-top” technique with the labrum left in situ when possible. In cases of larger rim resections, the labrum was first detached prior to rim resection and later re-attached using suture anchors. Cartilage lesions were either debrided or microfractured, depending on the lesion size and type. Cam morphologies were resected under the guidance of intraoperative fluoroscopy to assess the correct reshaping of the femoral head-neck junction. Postoperatively, patients were allowed free ROM and full weight-bearing with the use of crutches for 4 weeks. Patients were prescribed non-steroidal anti-inflammatory drugs (NSAIDs) for the first month postoperatively to minimise the risk of heterotopic ossification [26]. Antibiotic prophylaxis was not routinely used. Physiotherapy was initiated directly postoperatively and the protocol included exercises focusing on ROM, strength, endurance, balance and co-ordination. The intensity was gradually increased as tolerated by the patient under the guidance of a physiotherapist.

### Statistical analysis

With a clinically relevant change in iHOT-12 score of 10 points, a SD of 21 points (based on data from a previous study [21]) and an α-value of 0.05, the sample size calculation revealed that a power of > 90% would be reached with 75 patients. Statistical analysis was performed using version 9 of the SAS System for Windows. Descriptive statistics were used for patient demographics. Descriptive data were reported as the mean, median, SD and range. Categorical variables were tabulated with absolute and relative frequencies. The Wilcoxon signed rank test was used to compare preoperative PROM values with those obtained at the 5-year follow-up. Survivorship was calculated as the number of patients not undergoing THA at the 5-year follow-up divided by the number of patients at risk. All significance tests were two-sided and conducted at the 5% significance level.

## Results

**Fig. 1:**
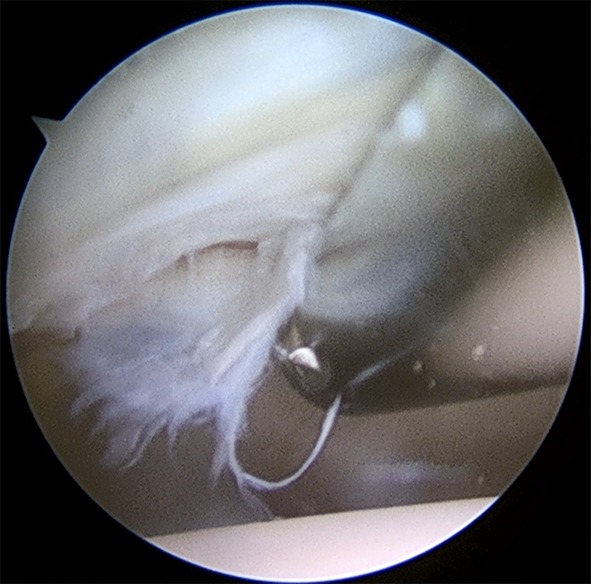
Perioperative visualisation of the hip joint. Example of Konan type 3a cartilage damage to the left hip, delamination of cartilage less than one-third of the distance from the acetabular rim to the cotyloid fossa

Of the included hips, 74 were isolated cam morphology (41.1%), 2 were isolated pincer morphology (1.1%) and 104 had both cam and pincer morphology (57.8%). During the follow-up period, four re-operations were performed following index surgery (2.2%). The performed procedures are shown in Table 2. Chondral damage was observed in 87 hips (65.4 per cent of the visualised hips). The most common type of chondral damage was Konan type 2, a tear between the labrum and the acetabular cartilage. Sixteen hips had bare bone in the acetabulum (12.0% of the visualised hips). The incidence of cartilage damage is shown in Table 3. Figure 1 depicts Konan 3a cartilage damage.

**Table 2:** Arthroscopic procedures performed

Procedure	Hips (%)
Cam	74 (41.1)
Pincer	2 (1.1)
Cam + pincer (combined)	104 (57.8)
Labral suture	19 (10.6)
Microfracture	13 (7.2)
Labral resection	13 (7,2)
Teres ligament resection	1 (0.6)

**Table 3:** Incidence of cartilage damage, classification according to Konan et al

Cartilage damage classification	Hips (% of visualised hips)
0	46 (34.6)
1a	10 (7.5)
1b	2 (1.5)
1c	0 (0.0)
2	33 (24.8)
3a	21 (15.8)
3b	4 (3.0)
3c	1 (0.8)
4a	9 (6.8)
4b	4 (3.0)
4c	3 (2.3)
Not visualised	21 (13.6% of hips)

A comparison between preoperative PROM scores and those obtained at the 5-year follow-up revealed statistically significant improvements for all outcome scores (*p* < 0.05), apart from the HSAS that was unchanged. Results and statistics are described in Table 4. At the 5-year-follow-up, 154 patients reported that they were satisfied with surgery (84.6%). Survivorship at the 5-year follow-up was 86.4%.

**Table 4:** Outcome scores

Outcome	Preoperative, mean (SD)	60 months, mean (SD)	Change	*p* value
iHOT-12	42.9 (18.3)	67.2 (27.5)	24.6 (25.7)	< 0.0001
HAGOS—symptoms	50.2 (17.8)	69.6 (24.0)	19.3 (22.6)	< 0.0001
HAGOS—pain	55.7 (19.5)	76.1 (23.4)	20.3 (24.0)	< 0.0001
HAGOS—daily activity	59.2 (24.7)	72.3 (31.1)	13.0 (30.6)	< 0.0001
HAGOS—sport	41.1 (22.1)	66.4 (29.9)	25.3 (30.5)	< 0.0001
HAGOS—physical activity	30.8 (28.2)	60.2 (33.1)	29.2 (38.6)	< 0.0001
HAGOS—quality of life	31.6 (18.4)	60.4 (29.6)	28.9 (28.7)	< 0.0001
EQ-5D	0.570 (0.296)	0.742 (0.292)	0.174 (0.354)	< 0.0001
EQ-VAS	66.6 (19.7)	74.4 (18.1)	8.03 (22.09)	< 0.0001
HSAS	3.13 (2.99)	3.17 (1.90)	−0.053 (3.021)	n.s
VAS—overall hip function	47.9 (20.6)	69.2 (25.6)	21.4 (28.0)	< 0.0001

*SD* Standard deviation, *iHOT-12* International Hip Outcome Tool, *VAS* visual analogue scale, *HAGOS* Copenhagen Hip and Groin Outcome Score, *EQ-5D* EuroQoL-5 Dimension Questionnaire, *HSAS* Hip Sports Activity Scale, *n.s*. non-significant

## Discussion

The key finding in present study is that outcomes at the 5-year follow-up after arthroscopic treatment for FAI syndrome demonstrated a significant improvement for all PROMs except the HSAS. The improvement exceeded the minimally important change (MIC) previously reported for the iHOT-12 and HAGOS, also indicating a clinically relevant improvement [21, 22]. In addition, 84.6% of patients reported that they were satisfied with surgery. Survivorship at the 5-year follow-up was 86.4%.

The main strengths of the present study are the prospective study design, large patient group and long follow-up period. An additional strength is the use of multiple modern PROMs which have been validated for use in young and active patients. Despite good overall results, the HSAS level was more or less unchanged at follow-up. The reason why patients maintained the same level of physical activity even though their hip function improved may have many explanations, such as social factors and/or lifestyle changes. Another explanation could be that patients performed at a high level of physical activity prior to surgery, despite experiencing pain, which is common in athletes [27]. Several studies have reported favourable short- to mid-term results following arthroscopic treatment for FAI syndrome [6, 7, 10]. The long-term outcomes are, however, less well understood. The 5-year outcome following arthroscopic treatment for FAI syndrome was recently reported by Hufeland et al., where the modified Harris Hip Score (mHHS) improved from 67.2 points to 86.4 points, with a 10.8% conversion rate to THA [14]. The mHHS is, however, based on a score initially constructed for use in elderly patients who had undergone THA and it is thus less well suited for use in young and active patients undergoing arthroscopic hip surgery [28, 29]. A study by Degen et al., including, but not limited to, patients with a 5-year follow-up, revealed comparable improvements in the mHHS, the Hip Outcome Score (HOS) and the iHOT-33 following arthroscopic treatment for FAI syndrome. The results were similar for both adolescents and non-adolescents [30]. For patients with preserved joint space (> 2 mm), Skendzel et al. reported a 16% conversion rate to THA within 5 years following arthroscopic surgery for FAI syndrome [31]. The use of different outcome scores in the present study limits the opportunity for comparison with earlier studies, as the iHOT and HAGOS have been recommended as preferred outcome scores when evaluating treatment for FAI syndrome [32]. Nevertheless, the conversion rate to THA in the present study was comparable to that in previous studies. The 2-year outcome for the present cohort has previously been published and the patient-reported outcomes were similar to those obtained at the 5-year follow-up, indicating stability in outcome over time [10]. The 5-year patient-reported outcomes in the present study were also comparable to the 2-year outcomes reported from the Danish Hip Arthroscopy Registry (DHAR), except for the HSAS, for which a smaller yet significant improvement was reported in the DHAR [7]. This difference could be due to a lower preoperative level of physical activity in the DHAR compared with the present study. The conversion rate to THA was also considerably lower in the DHAR, 0.8% vs. 13.6%, and this difference could be due to the longer follow-up period in the present study.

There are a number of limitations to the present study. As it is a cohort study, the lack of a control group makes it impossible to exclude any placebo effect on the results, or an effect independent of the treatment. However, long symptom duration prior to surgery should limit a return to the mean effect. Another limitation is the lack of recording of objective radiographic values, such as the alpha angle [33] for cam morphology and the lateral centre edge (LCE) angle [34] for pincer morphology. This reduces the opportunity for comparisons with other studies. However, it is important to remember that the diagnosis of FAI syndrome is not solely based on specific radiographic cutoff values but rather a mixture of radiographic findings, patient symptoms and clinical observations. Furthermore, pincer morphology is difficult to define radiographically [35]. The number of re-operations and conversions to THA were assessed from patient notes at the clinic where the primary surgery was performed. As a result, re-operations and conversions to THA performed at other clinics are not accounted for. However, we are confident that we provide our patients with a thorough follow-up.

## Conclusion

Arthroscopic treatment for FAI syndrome can be regarded as a viable treatment option, as promising PROM scores are reported at the 5-year follow-up; comparable to the results obtained at the 2-year follow-up. Future studies with longer outcomes including radiographic evaluation will also have the potential to reveal whether arthroscopic treatment for FAI syndrome has the potential to prevent the development of OA.
